# Naringin from sweet orange peel improves testicular function in high fat diet-induced diabetic rats by modulating xanthine oxidase/uric acid signaling and maintaining redox balance

**DOI:** 10.1186/s42826-024-00188-5

**Published:** 2024-02-18

**Authors:** Kazeem Bidemi Okesina, Adeyemi Fatai Odetayo, Wale Johnson Adeyemi, Ayodeji Johnson Ajibare, Akeem Ayodeji Okesina, Luqman Aribidesi Olayaki

**Affiliations:** 1 Department of Medical Physiology, School of Medicine and Pharmacy, College of Medicine and Health Sciences, University of Rwanda, Kigali, Rwanda; 2 Department of Physiology, Federal University of Health Sciences, Ila Orangun, Nigeria; 3 Department of Physiology, Adeleke University, Ede, Nigeria; 4 Department of Physiology, Lead City University, Ibadan, Nigeria; 5 Department of Clinical Medicine and Community Health, School of Health Sciences, College of Medicine and Health Sciences, University of Rwanda, Kigali, Rwanda; 6 Department of Physiology, University of Ilorin, Ilorin, Nigeria

**Keywords:** Testicular dysfunction, Xanthine oxidase/uric acid signaling, Redox balance, Diabetes mellitus, Naringin, Orange peel ethanolic extract

## Abstract

**Background:**

Type 2 diabetes mellitus (T2DM) is a metabolic disorder affecting many organs, including the testis. Naringin from orange peel extract (OPE) is a flavanone with fertility-enhancing properties. Hence, this study was designed to establish the effect of naringin on T2DM-induced testicular dysfunction. Thirty male (30) Wistar rats were randomized into five groups control, diabetes, diabetes + naringin, diabetes + OPE, and diabetes + metformin. The administrations were via the oral route and lasted for 28 days.

**Results:**

Naringin ameliorated T2DM-induced increase in FBS and decrease in serum insulin. It also abrogated T2DM-induced decrease in sperm quality, gonadotropin-releasing hormone, luteinizing hormone, follicle-stimulating hormone, testosterone, estradiol, prolactin, catalase, superoxide dismutase, and total antioxidant capacity. Furthermore, naringin prevented a T2DM-induced increase in malonaldehyde, tumor necrosis factor-alpha, C-reactive protein, xanthine oxidase (XO), and uric acid (UA), it was accompanied by the restoration of normal testicular histoarchitecture.

**Conclusions:**

Naringin prevented T2DM-induced testicular dysfunction by modulating XO/UA and restoring redox balance. Also, while the animals treated with OPE exhibited better ameliorative effects than their counterparts treated with naringin, the findings from this study showed that naringin would be a promising supplement for treating T2DM-induced male infertility.

## Background

Type 2 Diabetes mellitus (T2DM) is one of the commonest metabolic disorders characterized by impaired glucose metabolism. Statistically, about 537 million are suffering from diabetes mellitus (DM), which is estimated to increase to 643 million by 2030 [1]. An increase in the number of T2DM prevalence among people aged 10–18 years has been reported [2], indicating that T2DM sets in even before the desire to father a child. Mounting evidence has shown that DM and testicular dysfunctions are closely related [3, 4]. Compared with healthy individuals, impaired spermatogenesis and declined circulatory testosterone have been observed in humans suffering from DM [3, 5].

T2DM can impair testicular functions via direct testicular damage by distorting testicular redox balance via excessive reactive oxygen species (ROS) production [2]. However, the mechanisms associated with oxidative testicular damage have not been fully explored. Although xanthine oxidase (XO)/uric acid (UA) signaling has been shown to disrupt  testicular functions [6–8], its role in T2DM-induced testicular toxicity has not been fully established.

The XO/UA pathway is an inducer of redox imbalance [9]. XO is an enzyme responsible for catalyzing the conversion of hypoxanthine to xanthine and later to UA. During this process, oxygen molecules are used as an electron acceptor, which can lead to the generation of superoxide anion and other ROS. Although UA can be an antioxidant, its excessive production promotes oxidative stress and inflammation [10].

The existing anti-diabetic drugs are associated with multiple side effects; hence, it is suggested to identify a novel therapeutic agent for the treatment of diabetes that would be safer and more effective with a minimum of side effects. Ethnomedicines (pools of small molecules) could be a good source of novel drug identification. Citrus fruits and juices are an important source of bioactive compounds, including antioxidants such as naringin, ascorbic acid, flavonoids, phenolic compounds, and pectins. The traditional use of citrus fruits by the Persians have been documented [11]. The sweet orange (*Citrus sinensis*) (WFO, 2023) peel possesses the bulk of the citrus' health benefits and naringin is one of the most active ingredients in sweet orange. Others include narirutin, hesperidin, didymin, narringenin, and so on.

Naringin is a nontoxic flavone naturally found in orange peel extract (OPE) [12]. Naringin has been shown to have several activities, such as antidiabetic [12], antioxidant [13], and anti-inflammatory [14]. In fact, the ameliorative effect of naringin on sunscreen ingredients (such as TiO2)-induced toxicity has been associated with its antioxidant properties [15]. Furthermore, the findings that naringin scavenges free radicals generated by UV radiation further substantiates its antioxidant activities [16]. Despite these interesting findings, no study has associated the anti-oxidative properties of naringin with its possible modulatory activities on XO/UA signaling. In addition, the gonadoprotective effect of naringin has been established. Naringin has been shown to improve sperm quality [17] and testosterone synthesis [18]. However, the role of XO/UA in the gonadoprotective activities of naringin is not known. Hence, this study was designed to assess the effect of naringin on T2DM-induced testicular dysfunction. Also, the role of XO/UA signaling in T2DM-induced gonadotoxicity and possible modulatory role of naringin were established.

## Methods

### Chemicals

Naringin was obtained from Santa Cruz Biotechnology, TX, USA, while other chemicals used in this study, except otherwise stated, were purchased from Sigma-Aldrich, US.

### Extract Preparation

Sweet orange (*Citrus sinensis*) used for this study was obtained from Ilorin and the plant name was confirmed from World Flora Online (www.worldfloraonline.org). The samples were identified at the University of Ilorin Herbarium, Department of Plant Biology, with a Voucher No. UIH0001/159. The OPE extract was obtained as previously documented [19, 20]. Briefly, the oranges were washed before the peel was separated from the edible parts of the fruit. Thereafter, the peels were air dried for 4 weeks and then powdered by blending. The resultant (about 500 g) was subjected to cold extraction with 95% ethanol (4.5 L) for two days. The extract was filtered through Whatman No. 1 filter paper (Tokyo, Japan), and the extraction solvent was removed with an evaporator (Eyela N-1000, Tokyo Rikakikai Co., Tokyo, Japan) at 40^O^ C. The dry extract was re-dissolved in normal saline to a concentration of 50 mg/mL and kept at 20^O^ C until use.

### Animal and treatment

Thirty (30) adult male Wistar rats (180–200 g) were used for this study. The animals were housed under the natural condition of 12 h light/darkness cycle, and two weeks of acclimatization was allowed. The animals were later randomized into five (5) 28 days treatment groups as follows: Control, diabetic untreated (DMU), diabetic rats treated with 50 mg/kg of naringin (DM + naringin), diabetic rats treated with 600 mg/kg of OPE (DM + OPE), and diabetic rats treated with 180 mg/kg of metformin (DM + MET). The dosage of metformin and OPE used in this study is similar to what was used and reported by Olayaki et al. [20] and Adeyemi et al. [19] while naringin is similar with the dosage used by Murunga et al. [12] and Mahmoud et al. [21]. In addition, the dosage of metformin is below the earlier reported No Observable Adverse Effect Level (NOAEL) of 20 mg/kg by Sarmiento-Ortega et al. [22] and Zhang et al., [23].

### T2DM Induction

T2DM was induced after the 2 weeks of acclimatization as previously described [20]. The High-fat diet (HFD) with a low dose of streptozocin (35 mg/kg) diabetic induction was adopted because it closely mimics T2DM in human [21]. The composition of the HFD obtained from Olorunsogo Feed in Ilorin, Kwara State (maize = 5.5 kg, wheat = 0.5 kg, ground nut cake = 5.5 kg, soya meal/cake/full fat = 12.5 kg, palm kernel cake = 5.0 kg, bone meal = 0.5 kg, methionine = 0.25, lysine = 0.25) is similar with what was previously reported and used by Olayaki et al. [20].

### Sample collection

Overnight fasted rats were sacrificed via IP administration of 40 mg/kg of ketamine and 4 mg/kg of xylazine [24]. Blood samples were collected via cardiac puncture, emptied into plain bottles, and centrifuged at 5000 rpm for 15 min to obtain serum. Also, the left testes were harvested and preserved for tissue homogenate using phosphate buffer solution, while the right testes were preserved for histology using bouin solution.

### Biochemical analysis

#### Oral glucose tolerance test, FBS and serum insulin

The animals received oral administration of D-glucose solution (2 g/kg b.w.) following 12–14 h of overnight fasting. Blood glucose levels were determined at 0 min (before glucose loading), 30, 60, 90, and 120 min after oral glucose administration. The glucose levels and terminal FBS were measured using a digital glucometer (On Call®Plus ACON Laboratories, Inc. San Diego, CA, USA). Serum insulin level was estimated by Enzyme-Linked Immunosorbent Assay (ELISA) technique based on the manufacturer's guideline (RayBio®, GA, USA).

#### Semen analysis

Semen samples were obtained from the caudal epididymis, and the volume was estimated in a calibrated measuring cylinder using a densitometer. Sperm cells were counted by a hemocytometer using an improved Neubauer (Deep 1/10 mm, LABART, Germany) chamber Sperm morphology and percentage viability assay were determined from a total count of 400 spermatozoa in smears obtained with Wells and Awa stains (0.2 g of Eosin and 0.6g of Fast green dissolved in distilled water and ethanol in ratio 2:1) [25]. Sperm viability was determined using 1% Eosin and 5% Nigrosin in a 3% sodium citrate dehydrate solution as previously established [26].

#### Reproductive hormones

The serum level of gonadotropin-releasing hormone (GnRH) (Melsin, China), follicle-stimulating hormone (FSH), luteinizing hormone (LH), testosterone, estradiol, and prolactin (Bio-Inteco, UK) were estimated using ELISA method.

#### Testicular injury markers

Testicular lactate dehydrogenase (LDH) and alkaline phosphatase (ALP) (Aggape Diagnostic, Switzerland), and lactate (Abcam, China) were assayed by spectrophotometric method using ELISA kits.

#### Histology

Testicular histology was performed based on the established method with little modifications [27]. Briefly, the testis was fixed in bouin solution, dehydrated with ethanol series, cleared with toluene, embedded at room temperature, and blocked in paraffin wax. Hematoxylin and Eosin (H&E) stain was applied to the 5 µm thick paraffin sections of the testes.

#### Oxidative stress markers

Total antioxidant capacity (TAC) was assayed using a colorimetry method (Fortress Diagnostic Kit, Switzerland). Testicular malondialdehyde (MDA) was determined as previously documented [28]. Testicular superoxide dismutase (SOD) and catalase (CAT) were determined as previously established [8, 29].

#### Inflammatory markers

Testicular tumor necrotic factor-alpha (TNF-α) (Solarbio, China) and C-reactive protein (CRP) (Elabscience, USA) were assayed using an ELISA kit.

#### XO and UA

Testicular XO activities were determined as previously described [6], while UA concentration was determined using colorimetric methods (Precision, UK) using a spectrophotometer.

### Statistical analysis

The groups were analyzed with Graph-pad Prism version 9 for statistical comparison (*p* < 0.05) using one-way analysis of variance (ANOVA) followed by post hoc Tukey tests. All data were expressed as mean ± SEM, n = 6.

## Results

As shown in Table 1, all animals experienced a significant increase (*p* < 0.05) in blood glucose levels after 30 min of the oral glucose loading compared to basal glucose (0 min). Only animals in the standard control group recovered from the increased glucose level after 90 min. The remaining groups, except diabetic untreated (DM-Uii), had no significant difference in blood glucose level at 120 min when compared with the basal (0 min) blood glucose level.

**Table 1:** Effects of naringin on oral glucose tolerance test (OGTT)

Groups(mmol/L)	0 min	30 min	60 min	90 min	120 min
Control	5.91 ± 0.15	10.65 ± 0.83*	8.03 ± 0.31*	6.49 ± 0.32	6.03 ± 0.29
DMU	17.02 ± 0.50	28.25 ± 2.12*	26.99 ± 1.21*	25.04 ± 1.32*	25.64 ± 0.72*
DM + Naringin	11.04 ± 0.80	18.41 ± 0.73*	19.97 ± 0.52*	16.53 ± 0.48*	12.54 ± 0.43
DM + OPE	7.24 ± 0.21	17.63 ± 0.25*	16.03 ± 0.31*	14.79 ± 0.32*	7.84 ± 0.21
DM + Met	7.02 ± 0.41	20.06 ± 0.42*	11.94 ± 0.43*	10.01 ± 0.42*	7.32 ± 0.21

Data were analyzed by one way ANOVA (expressed as mean ± SEM) and Tukey’s posthoc test. The level of significance compared with the basal was determined at *p < 0.05. Control (normal saline)

DMU: Diabetic untreated DM + Naringin: Diabetic treated with naringin; DM + OPE: Diabetic, treated with orange peel; DM + Met: Diabetic treated with metformin

**Fig. 1:**
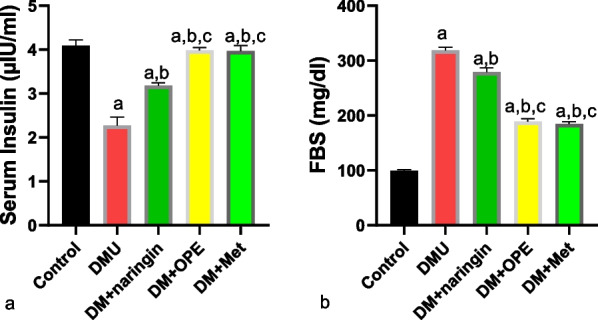
Effect of naringin on **a** serum insulin **b** fasting blood sugar (FBS). ^a^*P* < 0.05 vs control, ^b^*P* < 0.05 vs DMU, ^c^*P* < 0.05 vs DM + naringin, ^d^*P* < 0.05 vs DM + OPE. Data were analyzed by one way ANOVA (and expressed as mean ± SEM) followed Tukey’s posthoc test. DMU: Diabetic untreated, DM + naringin: Diabetic treated with naringin; DM + OPE: Diabetic treated with orange peel extract; DM + met: Diabetic, treated with metformin

As shown in Fig. 1, naringin ameliorated the T2DM-induced decrease in serum insulin and increased FBS. Although naringin prevented T2DM-induced hyperglycemia and glucose dysmetabolism, a better ameliorative effect was observed in the animals treated with either OPE or metformin.

Furthermore, reduced sperm volume, motility, count, viability, and normal morphology were observed in the diabetic untreated rats compared with their counterparts in the control group (Table 2). These observed impairments in sperm quality were abrogated in rats treated with naringin. In addition, the observed increase in the tail, head, and neck defect following T2DM induction compared with the control group was significantly reduced by naringin treatment (Table 3). While naringin prevented the observed impairment in sperm quality following T2DM induction, a better ameliorative effect was observed in the animals treated with OPE.

**Table 2:** Effects of naringin on semen analysis

Sperm analysis	Groups				
	Control	DMU	DM + Naringin	DM + OPE	DM + Met
Volume (ml)	2.33 ± 0.22	1.23 ± .0.15^a^	1.85 ± 0.17^a,b^	2.25 ± .0.21^b,c^	2.29 ± 0.25^b,c^
%Motility	76.00 ± 4.76	53.25 ± 4.15^a^	60.25 ± 9.36^a,b^	68.25 ± 4.13^a,b,c^	69.80 ± 6.94^a,b,c^
Sperm count (*10^6^)/ml	7.03 ± 5.48	5.75 ± 4.77^a^	6.98 ± 7.05^a,b^	7.05 ± 2.75^a,b^	7.01 ± 4.71^a,b^
Sperm Viability	92.00 ± 6.62	68.50 ± 3.18^a^	79.50 ± 3.88^a,b^	82.75 ± 6.01^a,b^	80.20 ± 3.06^a,b^

Data were analyzed by one way ANOVA (and expressed as mean ± SEM) and Tukey’s posthoc test. DMU: Diabetic untreated, DM + naringin: Diabetic treated with naringin; DM + OPE: Diabetic treated with orange peel extract; DM + met: Diabetic, treated with metformin

^a^*P* < 0.05 vs control

^b^*P* < 0.05 vs DMU

^c^*P* < 0.05 vs DM + naringin

^d^*P* < 0.05 vs DM + OPE

**Table 3:** Effects of naringin on sperm morphology

Sperm analysis	Groups				
	Control	DMU	DM + Naringin	DM + OPE	DM + Met
Normal Morphology	72.00 ± 2.62	45.25 ± 5.51^a^	55.00 ± 3.76^a,b^	63.75 ± 6.01^a,b,c^	65.20 ± 3.06^a,b,c^
Tail defect	10.33 ± 1.67	26.00 ± 2.48^a^	19.50 ± 2.26^a,b^	17.00 ± 2.84^a,b,c^	16.80 ± 1.59^a,b,c^
Head defect	12.83 ± 1.01	16.75 ± 1.25^a^	14.25 ± 1.49^a,b^	12.25 ± 1.97^b,c^	10.40 ± 1.33^a,b,c^
Neck defect	6.83 ± 0.48	11.50 ± 0.50^a^	11.05 ± 0.63^a^	6.75 ± 1.38^b,c^	8.60 ± 0.81^a,b,c,d^

Data were analyzed by one way ANOVA(and expressed as mean ± SEM) followed Tukey’s posthoc test. DMU: Diabetic untreated, DM + naringin: Diabetic treated with naringin; DM + OPE: Diabetic treated with orange peel extract; DM + met: Diabetic, treated with metformin

^a^*P* < 0.05 vs control

^b^*P* < 0.05 vs DMU

^c^*P* < 0.05 vs DM + naringin

^d^*P* < 0.05 vs DM + OPE

Also, a significant decrease in serum GnRH, LH, FSH, and testosterone and increased estradiol and prolactin was observed in diabetic untreated animals compared with the control (Table 4). This observed T2DM-induced hormonal imbalance was ameliorated by naringin and OPE. Although naringin and OPE prevented the observed hormonal imbalance, animals treated with OPE exhibited a better ameliorative effect in all the parameters except in estradiol and prolactin, where there was no significant difference between the two groups.

**Table 4:** Effects of naringin on reproductive hormones

	CTRL	DMU	DM + naringin	DM + OPE	DM + Met
GnRH(mIU/mL)	11.32 ± 0.61	4.23 ± 0.31^a^	6.12.4 ± 0.31^a,b^	7.29 ± 0.29^a,b,c^	7.31 ± 0.52^a,b,c^
FSH (mIU/ml)	3.98 ± 0.23	1.93 ± 0.20^a^	2.32 ± 0.43^a,b^	2.99 ± 0.32^a,b,c^	2.96 ± 0.39^a,b,c,^
LH (mIU/ml)	6.94 ± 0.24	1.52 ± 0.32^a^	4.07 ± 0.35^a,b^	5.43 ± 0.45^a,b,c^	5.55 ± 0.45^a,b,c^
Testosterone (ng/ml)	3.66 ± 0.31	0.75 ± 0.25^a^	1.34 ± 0.34^a,b^	1.57 ± 0.31^a,b,c^	1.56 ± 0.21^a,b,c^
Estradiol (pg/ml)	0.57 ± 0.13	2.64 ± 0.23^a^	0.61 ± 0.25^b^	0.60 ± 0.32^b^	0.59 ± 0.36^b^
Prolactin (ng/ml)	0.83 ± 0.21	3.15 ± 0.36^a^	0.82 ± 0.16^b^	0.82 ± 0.21^b^	0.81 ± 0.27^b^

Data were analyzed by one way ANOVA (and expressed as mean ± SEM) followed Tukey’s posthoc test. DMU: Diabetic untreated, DM + naringin: Diabetic treated with naringin; DM + OPE: Diabetic treated with orange peel extract; DM + met: Diabetic, treated with metformin

^a^*P* < 0.05 vs control

^b^*P* < 0.05 vs DMU

^c^*P* < 0.05 vs DM + naringin

^d^*P* < 0.05 vs DM + OPE

**Fig. 2:**
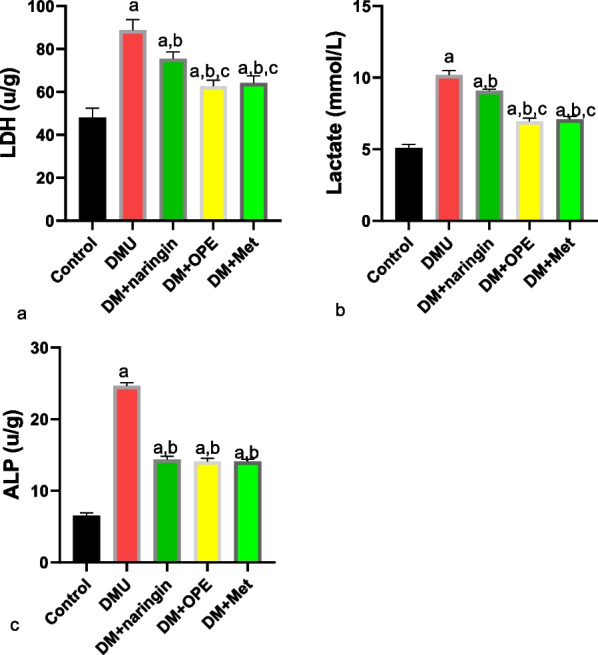
Effect of naringin on testicular **a** lactate dehydrogenase (LDH) **b** lactate **c** alkaline phosphatase (ALP). ^a^*P* < 0.05 vs control, ^*b*^*P* < 0.05 vs DMU, ^c^*P* < 0.05 vs DM + naringin, ^d^*P* < 0.05 vs DM + OPE. Data were analyzed by one way ANOVA (and expressed as mean ± SEM) followed Tukey’s posthoc test. DMU: Diabetic untreated, DM + naringin: Diabetic treated with naringin; DM + OPE: Diabetic treated with orange peel extract; DM + met: Diabetic, treated with metformin

As shown in Fig. 2, T2DM significantly increased testicular LDH, lactate, and ALP compared to the control group animals. These observed increased were significantly reduced in the animals treated with naringin, OPE, and metformin. While there was a significant decrease in testicular injury markers following naringin treatment, the observed decrease in these markers were more pronounced in the OPE and metformin groups except in testicular ALP, where there was no significant difference between the three groups.

**Fig. 3:**
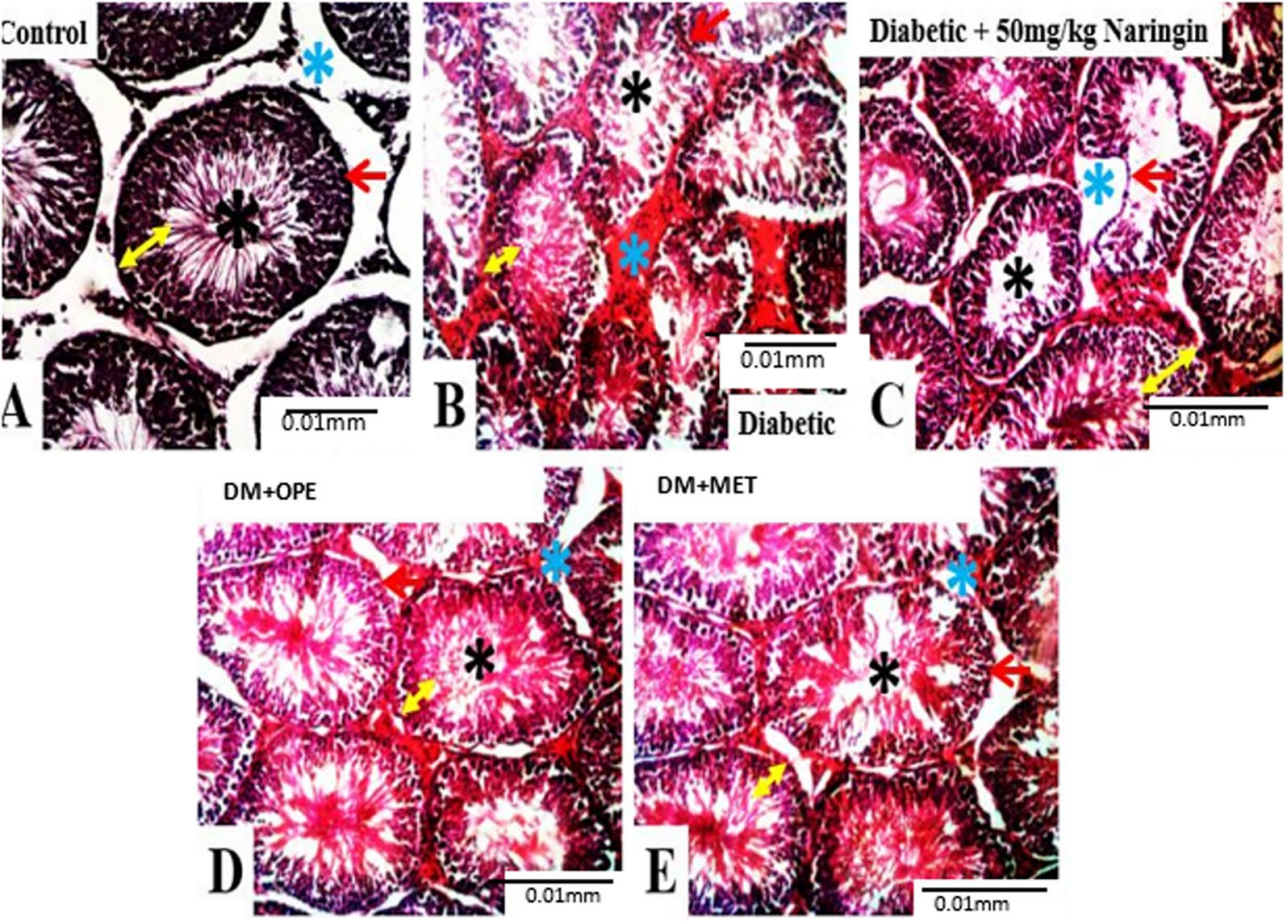
Histology of the Testes; Stain H and E; × 100. Lumen of seminiferous tubule (black star), basement membrane (red arrow), interstitial space (blue star), spermatogenic cells (yellow spanned arrow). The testicular histoarchitecture in the control group (**A**) showed a round-oval-shaped seminiferous tubule with an intact basement membrane containing proliferating spermatogenic cells; there is also the presence of testosterone-secreting Leydig cells within the interstitial spaces separating the seminiferous tubules. Diabetic animals (**B**) showed disrupted testicular morphology, abnormal shape of the seminiferous tubule with thin basement membrane, degeneration of appreciable sum of spermatogenic cells, presence of few or no Leydig cells within the interstitial spaces, and reduced spermatogenesis rate. Diabetic + naringin (**C**) showed a slightly improved morphology of the seminiferous tubule and spermatogenesis rate. DM + OPE (**D**) and DM + Met (**E**) showed oval seminiferous tubules with improved proliferation of spermatogenic cells

Furthermore, the testicular histoarchitecture in the control group (A) showed a round-oval-shaped seminiferous tubule with an intact basement membrane containing proliferating spermatogenic cells; there is also the presence of testosterone-secreting Leydig cells within the interstitial spaces separating the seminiferous tubules (Fig. 3). Diabetic animals (B) showed disrupted testicular morphology, abnormal shape of the seminiferous tubule with thin basement membrane, degeneration of appreciable sum of spermatogenic cells, presence of few or no Leydig cells within the interstitial spaces, and reduced spermatogenesis rate. Diabetic animals treated with 100 mg/kg b.w. naringin (C) showed a slightly improved morphology of the seminiferous tubule and spermatogenesis rate compared to untreated diabetics (B). Diabetic animals treated with 600 mg/kg b.w. of OPE and metformin (D and E, respectively) showed oval seminiferous tubules with improved proliferation of spermatogenic cells.

Testicular MDA was significantly increased in diabetic untreated animals compared with the control (Table 5). This observed increase was ameliorated in animals treated with naringin, OPE, and metformin. In addition, the observed decrease in testicular SOD, CAT, and TAC following diabetic induction was abrogated in the animals treated with naringin, OPE, and SOD. Although naringin prevented T2DM-induced oxidative stress, animals treated with OPE and metformin exhibited a better ameliorative effect.

**Table 5:** Effect of naringin on oxidative stress and inflammatory markers

	CTRL	DMU	DM + naringin	DM + OPE	DM + Met
MDA (µM)	1.59 ± 0.10	8.98 ± 0.18^a^	5.26 ± 0.16^a,b^	3.56 ± 0.15^a,b,c^	2.69 ± 1.70^a,b,c,d^
SOD (U/mg)	4.25 ± 0.16	1.13 ± 0.10^a^	2.32 ± 0.12^a,b^	3.21 ± 0.18^a,b,c^	3.11 ± 0.13^a,b,c,^
Catalase (U/mg)	15.94 ± 0.28	7.38 ± 0.27^a^	10.78 ± 0.39^a,b^	12.26 ± 0.31^a,b,c^	11.98 ± 0.41^a,b,c^
TAC(mmol/g tissue)	1.08 ± 0.03	0.15 ± 0.08^a^	0.79 ± 0.05^a,b^	0.91 ± 0.04^a,b,c^	0.93 ± 0.06^a,b,c^
TnF-a (pg/mL)	5.57 ± 0.13	10.89 ± 0.23^a^	7.98 ± 0.32^a,b^	6.42 ± 0.32 ^a,b,c^	6.51 ± 0.36^a,b,c^
CRP (ng/ml)	0.14 ± 0.01	0.63 ± 0.06^a^	0.48 ± 0.02^a,b^	0.27 ± 0.01^a,b,c^	0.25 ± 0.02^a,b,c^

Data were analyzed by one way ANOVA (and expressed as mean ± SEM) followed Tukey’s posthoc test. DMU: Diabetic untreated, DM + naringin: Diabetic treated with naringin; DM + OPE: Diabetic treated with orange peel extract; DM + met: Diabetic, treated with metformin

^a^*P* < 0.05 vs control

^b^*P* < 0.05 vs DMU

^c^*P* < 0.05 vs DM + naringin

^d^*P* < 0.05 vs DM + OPE

Also, the observed increase in TnF-α and CRP following diabetic induction was ameliorated by naringin, OPE, and metformin administration. However, a better ameliorative effect was observed in animals treated with OPE and metformin.

**Fig. 4:**
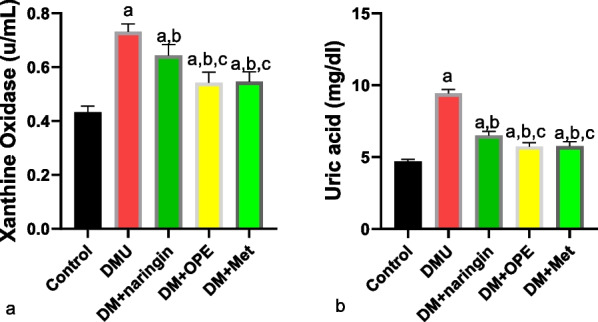
Effect of naringin on testicular **a** xanthine oxidase **b** uric acid. ^a^*P* < 0.05 vs control, ^b^*P* < 0.05 vs DMU, ^c^*P* < 0.05 vs DM + naringin, ^d^*P* < 0.05 vs DM + OPE. Data were analyzed by one way ANOVA (and expressed as mean ± SEM) followed Tukey’s posthoc test. DMU: Diabetic untreated, DM + naringin: Diabetic treated with naringin; DM + OPE: Diabetic treated with orange peel extract; DM + met: Diabetic, treated with metformin

In addition, testicular XO and UA were significantly increased following T2DM induction, while naringin, OPE, and metformin reversed the observed increase (Fig. 4). Although the T2DM-induced increase in XO and UA was reversed in all the treatment groups, a better ameliorative effect was observed in OPE and metformin-treated animals.

## Discussion

The surge and crash of blood glucose and serum insulin resulting from chronic T2DM can lead to an irreparable assault on different body organs, including the testis. Testis is the primary male reproductive organ and one of the target organs for T2DM [3]. The two major functions of the testis include sperm production (spermatogenesis) and testosterone production (steroidogenesis). In this study, the animals in the diabetic untreated group exhibited a significant decrease in sperm quality and circulating testosterone compared with their counterparts in the control group. These findings agreed with the study of Maresch et al. [3] and Ding et al. [30]. The T2DM-induced testicular dysfunction could result from hormonal imbalance (i.e., hormonal-dependent mechanism) or direct damage to the testis (non-hormonal-dependent mechanism).

Naringin and orange peel extract ameliorates T2DM-induced hormonal imbalance. Testicular functions are tightly regulated by the hypothalamic-pituitary–testicular (HPT) axis [26]. The hypothalamus secretes GnRH, which stimulates the pituitary gland to release LH and FSH. LH stimulates the testis to produce testosterone (steroidogenesis), while FSH stimulates Sertoli cells to produce sperm (spermatogenesis). Also, the testosterone produced from the testis assists in spermatogenesis [31]. The synthesized testosterone inhibits the pituitary gland's and hypothalamus's activities to maintain its optimal circulatory level [24]. This closed circuit is responsible for maintaining optimal testicular function, and any impairment affecting this circuit could impair male sexual function. Hence, this study's observed T2DM-induced hormonal imbalance could account for the observed impaired sperm quality in untreated diabetic animals.

Furthermore, naringin and orange peel abrogated T2DM-induced distortion in testicular histology and increase in testicular injury markers. The observed increase in testicular injury markers and impaired testicular histoarchitecture indicates the direct toxic effect of T2DM on testicular functions. Spermatogenesis is a highly regulated process, and energy imbalance disrupts various signaling responsible for regulating sperm production [32]. The observed T2DM-induced increase in LDH and lactate indicates an energy imbalance [33], suggesting an impaired spermatogenesis.

Furthermore, the observed increase in oxidative stress (evidenced by the increase in MDA and decrease in SOD, catalase, and TAC) and inflammatory (increase in CRP and TnF-α) markers could explain the observed testicular dysfunction following T2DM induction. Oxidative stress and inflammation contribute to testicular dysfunction [34]. Spermatogenesis and sperm quality are greatly affected by excess ROS [35]. Also, sustained excessive ROS can trigger cytokines overproduction leading to inflammation which may arrest sperm production and impair sperm quality [36].

Furthermore, T2DM impaired XO/UA signaling. T2DM is a major trigger for oxidative stress and the role of XO/UA in T2DM-mediated oxidative stress has not been fully established. Excess production of UA is a key player and primary cause of oxidative stress [37]. UA is produced via the purine synthesis pathway through the activities of xanthine oxidoreductase. Xanthine oxidoreductase is the enzyme responsible for the catalysis of the last two final steps of the purine system. It converts hypoxanthine to xanthine, which eventually leads to the production of UA [38]. Xanthine oxidoreductase exists as either xanthine dehydrogenase (XDH) or XO [39]. XDH reduces NAD + to NADH and can be reversibly or irreversible converted to XO in mammals [40]. XO, on the other hand, utilizes oxygen to generate hydrogen peroxide (H2O2) and superoxide ions [41]; aside from the production of H_2_O_2_ and superoxide from the activities of XO, the UA, which is the final product is also a pro-oxidant. Although UA acts as an antioxidant, it contributes to oxidative stress when produced in excess [6]. The observed increase in testicular XO and UA following T2DM suggests that XO/UA could be the primary mechanism responsible for the T2DM-induced oxide-inflammatory response observed in this study.

This study demonstrated that naringin from orange peel extract might ameliorate T2DM-induced testicular dysfunction via its antioxidant and anti-inflammatory activities, and XO/UA could be a target for the gonado-protective strategy of naringin. These findings agree with the study of Pengnet et al. [42], Adebiyi et al. [43], and Pengnet et al. [44], which reported that naringin (a major constituent of orange peel) suppressed oxido-inflammatory response by maintaining redox balance. In addition to a redox balance restoration, naringin restored the testicular histoarhitecture and function by preventing seminiferous tubular distortion, spermatogenic cell degeneration, and loss of Leydig cells within the interstitial spaces.

Although naringin prevented T2DM-induced testicular dysfunction, it is not as effective as OPE. This could be due to the synergistic activities of naringin with other components of OPE, such as vitamin C and naringenin [45, 46]. An unreported findings from our laboratory showed that 45.57 mg/100 mL of vitamin C is present in OPE, while the orange juice had 30.38 mg/100 mL of Vitamin C. Vitamin C is an antioxidant, and could work synergistically with naringin to mitigate the observed T2DM-induced testicular dysfunction. This could be the reason why the animals treated with OPEE exhibited better ameliorative effects than their counterparts treated with naringin.

## Conclusions

Naringin, a major bioactive flavonoid in sweet orange/citrus fruits, protects against T2DM by modulating the XO/UA signaling and maintaining redox balance. Thus, it exhibits interesting therapeutic potential for use as an effective alternative treatment for T2DM patients. However, this study was conducted on animals, and well-controlled trials will be necessary to elucidate the potential of naringin in clinical practice. Also, effort must be made to create a novel formulation to improve naringin bioavailability. Nevertheless, the ability of naringin to restore serum insulin, FBS, redox balance, and testicular functions following T2DM induction demonstrates its great potential to become an innovative and safe antidiabetic and fertility-enhancing drug.

## Data Availability

The data used for the study are available from the corresponding author upon request.

## Abbreviations

CAT: Catalase
CRP: C-reactive protein
FBS: Fasting blood sugar
FSH: Follicle stimulating hormone
LH: Luteinizing hormone
OPE: Orange peel extract
SOD: Super oxide dismutase
T2DM: Type 2 diabetic mellitus
TAC: Total antioxidant capacity
UA: Uric acid
XO: Xanthine oxidase
